# Enzymatically-Crosslinked Gelatin Hydrogels with Nanostructured Architecture and Self-Healing Performance for Potential Use as Wound Dressings

**DOI:** 10.3390/polym15030780

**Published:** 2023-02-03

**Authors:** Alina Gabriela Rusu, Loredana Elena Nita, Natalia Simionescu, Alina Ghilan, Aurica P. Chiriac, Liliana Mititelu-Tartau

**Affiliations:** 1 Natural Polymers, Bioactive and Biocompatible Materials Department, “Petru Poni” Institute of Chemistry, 41-A Grigore Ghica Voda Alley, 700487 Iasi, Romania; 2 Center of Advanced Research in Bionanoconjugates and Biopolymers, “Petru Poni” Institute of Chemistry, 41-A Grigore Ghica Voda Alley, 700487 Iasi, Romania; 3 Department of Pharmacology, Clinical Pharmacology and Algesiology, “Grigore T. Popa” University of Medicine and Pharmacy, Universitǎţii Street 16, 700115 Iasi, Romania

**Keywords:** transglutaminase, hydrogels, nanogels

## Abstract

Development of natural protein-based hydrogels with self-healing performance and tunable physical properties has attracted increased attention owing to their wide potential not only in the pharmaceutical field, but also in wounds management. This work reports the development of a versatile hydrogel based on enzymatically-crosslinked gelatin and nanogels loaded with amoxicillin (Amox), an antibiotic used in wound infections. The transglutaminase (TGase)-crosslinked hydrogels and encapsulating nanogels were formed rapidly through enzymatic crosslinking and self-assembly interactions in mild conditions. The nanogels formed through the self-assemble of maleoyl-chitosan (MAC5) and polyaspartic acid (PAS) may have positive influence on the self-healing capacity and drug distribution within the hydrogel network through the interactions established between gelatin and gel-like nanocarriers. The physicochemical properties of the enzymatically-crosslinked hydrogels, such as internal structure, swelling and degradation behavior, were studied. In addition, the Amox release studies indicated a rapid release when the pH of the medium decreased, which represents a favorable characteristic for use in the healing of infected wounds. It was further observed through the in vitro and in vivo biocompatibility assays that the optimized scaffolds have great potential to be used as wound dressings.

## 1. Introduction

In the field of soft tissue engineering, researchers have long sought to identify the characteristics of an ideal biomaterial that mimics the self-healing structure and composition of the extracellular matrix (ECM) and can stimulate cell organization and direct in vivo tissue regeneration [1]. In this regard, biopolymers-based hydrogels have emerged as an attractive class of materials, due to their outstanding resemblance to natural living tissues from the point of view of their structural properties and functionalities. As naturally-derived biomaterials, hyaluronic acid [2,3] and gelatin [4] are examples of hydrophilic polymers that have found widespread usage in biomedical applications due to their biocompatibility, biodegradability, and non-cytotoxicity. Moreover, hydrogels based on gelatin, a biocompatible product of collagen obtained through hydrolysis [5], which has a significant role in the proliferation of fibroblasts at wound sites, have been regarded as one of the most promising candidates for wound care.

Traditional gelatin hydrogels have, however, been used only in a limited number of biomedical applications due to their low mechanical qualities, rapid biodegradation and minimal antibacterial activity.

Therefore, to crosslink the amino and carboxyl groups of gelatin molecules, chemical crosslinking agents such as glutaraldehyde and carbodiimide have been used [6,7].

Over the years, methacryloyl gelatin electrospun 3D fibrous scaffolds (GelMA) with elasticity and tunable degradation rate have been successfully designed as biomaterials for tissue engineering or drug delivery [8,9]. Modification of gelatin with reactive methacryloyl groups endowed the electrospun gelatin fibers with the physicochemical adaptability of photo-crosslinkable materials required to aid wound healing. In skin regeneration applications, GelMA scaffolds have been shown to effectively aid wound regeneration by guiding cellular processes [10,11]. However, the potential cytotoxicity of chemical crosslinkers and photoinitiators has limited their use in biomedicine [12,13].

Current studies reported in the literature have investigated the potential cytotoxicity of various photoinitiators. Sabnis et al. investigated the effects of Irgacure 2959 photoinitiator and UV light exposure on human aortic smooth muscle cells (HASMCs) showing a cytotoxic dose-response correlated with only photoinitiator concentration. Additionally, it was demonstrated in their study that with the addition of 50 mg/L ascorbic acid, a radical scavenger, the cytotoxicity can be reduced and thus, in this case, the mechanism of toxicity may be related to the initial free radical generation [14]. Another photoinitiator that is commercially available and widely utilized in medical applications together with GelMA is lithium phenyl (2,4,6-trimethylbenzoyl) phosphinate (LAP). The primary concern which has been raised in recent years is its mutagenicity potential, especially considering the effect of light exposure during crosslinking. Nguyen et al. evaluated the cytotoxic and mutagenic effects of using LAP in combination with GelMA to produce a material for bioprinting [15]. The cytotoxicity of LAP on M-1 collecting duct cells increased following exposure to UV light using the necessary conditions for photopolymerization of GelMA with LAP (0.1 wt% LAP) and 10 min of 9.6 mW/cm^2^ light.

These limitations generated by crosslinking methods can be overcome by using enzymes to form covalently crosslinked hydrogels. Enzyme-mediated crosslinking by tyrosinases [16], transferases [17] or peroxidases [18] have proven their efficiency and therefore, they have attracted increasing attention for application in polymer hydrogel synthesis due to the environmentally-friendly process and the possibility of obtaining biomaterials with extracellular matrix-mimicking properties. Transglutaminase (TGase), a widely present enzyme in nature, has been demonstrated to catalyze the formation of isopeptide bonds between proteins by mimicking in vivo biosynthetic processes, which can significantly improve protein gelation [19,20].

Moreover, the necessity of hydrogels with the capacity to flow when shear stress is applied (i.e., shear-thinning) and reform after stress removal (i.e., self-healing) has grown in popularity as a means of overcoming the drawbacks of in situ crosslinking methods.

Furthermore, bottom-up approaches combining biocatalyst crosslinking with self-assembled nanomaterials manifesting secondary interactions such as hydrogen bonds, electrostatic or hydrophobic interactions responsible for the formation of reversible crosslinks, have led to the development of hydrogels with complex functionalities like self-healing capacity and architectures [21]. Inspired by living tissues, self-healing materials, through inductive reformation of covalent or noncovalent bonds, are capable of achieving the rebounding of cleaved or displaced macromolecular segments by reconstructing the damaged area after the application of a mechanical stress. In medicine and especially in wound care applications, the autonomously self-healing ability of wound dressings may be desirable, as it will be able to sustain tissue restoration while withstanding the main stages of wound healing (homeostasis, inflammation, proliferation, and maturation) which involve numerous transformative cellular processes [22,23,24].

Here we report a new concept for the fabrication of extracellular matrix-mimicking scaffolds with tunable physico-chemical properties and self-healing properties by employing enzymatically-crosslinked gelatin networks/embedded nanogels interactions. The novelty of this kind of nanostructured hydrogels consists in combining the TGase-mediated crosslinking of gelatin, a green approach, with self-assembled polyelectrolyte nanogels, which can act not only as building blocks but also as drug reservoirs capable of transporting their cargos and protecting them against in vivo degradation. Moreover, all the compounds used to obtain the systems investigated in this study are part of the biocompatible materials category. Through the selective adsorption of nanogels to crosslinked gelatin fibers, reversible interactions like hydrogen bonds are formed that can enable a smooth flow under applied shear stress, followed by rapid self-healing of the material when the applied stress is removed. Toward this, TGase-crosslinked gelatin scaffolds were embedded with amoxicillin-loaded (Amox) nanogels based on maleoyl-chitosan and polyaspartic acid. The inclusion of antibiotic-loaded nanogels also contributed to a more homogenous distribution of Amox within the polymer network and imprinted a pH-responsive drug delivery behavior to the new hydrogels. Notably, all components involved in hydrogels preparation are biocompatible with cell viability above 85%.

The tunable physico-chemical properties, including water retention capacity, biodegradability, drug delivery and self-healing ability as well as the in vitro and in vivo biocompatibility of the prepared hydrogels, make them promising biomaterials as therapeutic devices for wound care.

## 2. Materials and Methods

### 2.1. Materials

Gelatin, transglutaminase from guinea pig liver (≥1.5 units/mg protein) (TGase), collagenase, chitosan (Cs) (MW = 80 kDa), maleic anhydride (MA), 2,4,6-trinitrobenzenesulfonic acid solution (TNBS) (5% (*w*/*v*) in H_2_O), and amoxicillin (Amox) were all purchased from Sigma-Aldrich. Aspartic acid from Sigma-Aldrich and mesitylene and sulfolane from Fluka Chemika were used as precursor and organic solvents for poly(aspartic acid) (PAS) synthesis. Chemicals were used exactly as received and were all of analytical quality. An Ultra Clear TWF UV system was utilized to produce the deionized water that was used in several of the studies.

### 2.2. Hydrogels Preparation

For hydrogel preparation, a gelatin stock solution was prepared at a fixed concentration of 4% *w*/*v*. To prepare the TGase-crosslinked nanostructured hydrogels (HGel3 and HGel4) (Table 1), 5 mL gelatin, 1 mL maleoyl-chitosan (MAC5)/PAS nanogels and 0.5 mL TGase solution of various concentrations (2 U/g and 5 U/g) were well mixed, and the final solution was incubated at 37 °C for 2 h for crosslinking. Next, the enzymatically-crosslinked hydrogels were transferred to an oven at 70 °C for 10 min to deactivate the enzyme, freeze-dried, and stored at 4 °C until further analyses.

For comparative studies, similar method of preparation was used to obtain hydrogels based only on gelatin or gelatin and nanogels, without utilizing TGase for crosslinking (HGel1 and HGel2).

As previously described [25], the nanogels solution was made by combining the PAS and modified Cs solutions [MAC5/PAS ratio = 3.3/1(*w*/*w*)] while mixing the solutions until a slight iridescence appeared. Functionalization of Cs with MA [23] and the synthesis of PAS [26], both compounds used in nanogels formulation, were already published by our group.

Drug loading of nanogels was realized by initially dissolving Amox in MAC5 solution, over which PAS solution was added drop by drop, while mixing gently. The MAC5/PAS nanogels-to-Amox ratio utilized in this study for drug encapsulation was 2:1 (*w*/*w*) [25]. A thorough description of the mixture composition utilized to form hydrogels is provided in Table 1.

### 2.3. Fourier-Transform Infrared Spectroscopy (FT-IR)

FT-IR spectra of precursors and freeze-dried hydrogels were registered by a Vertex Bruker spectrophotometer (Ettlingen, Germany) in transmittance mode. The FTIR spectra of all compounds were acquired using the ATR technique in a wave number range of 4000–400 cm^−1^ and 64 scans with a resolution accuracy of 4 cm^−1^ at room temperature.

### 2.4. Scanning Electron Microscopy (SEM) Analysis

SEM technique was used to examine the morphologies in section of the hydrogels (10–12 mm diameter × 7–9 mm height), using a Quanta 200 instrument with EDAX–Elemental Analysis System operating with secondary electrons at 20 kV, under low vacuum mode (60–100 Pa) and LFD detector (from FEI Company, Hillsboro, OR, USA). Prior to the scanning process, the hydrogels were freeze-dried. In addition, before analysis, the samples were sputter-coated with gold. The average pore size and distribution of pores size were determined by using the ImageJ and Origin 8.5 software. For statistical analysis of SEM data, the one-way ANOVA test and Tukey test were utilized.

### 2.5. Degree of Crosslinking Determination

The degree of crosslinking of the gels was determined by TNBS assay [27,28]. Briefly, freeze-dried hydrogels were treated with a mixture of 1 mL of 0.5% solution of TNBS and 1 mL of 4% sodium bicarbonate at 60 °C for 4 h. The unreacted gelatin in the hydrogel reacts with TNBS and forms a soluble complex. An amount of 1 mL of this solution was further treated with 3 mL of 6 N HCl at 40 °C for 1.5 h and its absorbance was determined at 334 nm after dilution spectrophotometrically (Jenway 6305, Stone, Staffordshire, UK). The amount of free amine groups that were still present following the crosslinking procedure was calculated using the assumption that the non-crosslinked sample had 100% of the free amine groups that were available.

The crosslinking degree was calculated as follows [Equation (1)]:

(1) $$Crosslinking\;degree\%=1-\frac{Abs_{HGel1}}{Abs_{HGeli}}\times 100$$

where *Abs_HGel1_* is the absorbance of the HGel1 based only on non-crosslinked gelatin and *Abs_HGeli_* (*i* = 3 or 4) is the absorbance of TGAse crosslinked hydrogels (HGel3 or HGel4).

All the experiments were done in triplicate.

### 2.6. Swelling Capacity of Hydrogels

The swelling capacity of hydrogels was determined in buffer solutions via the gravimetric method. The water retention capacity over time was investigated through recording the swelling kinetics curves for all hydrogels at 37 °C, in buffer solution of pH 7.4, which simulates the conditions of internal physiological medium. Additionally, the hydrogels’ pH sensitivity was assessed by immersing a specific amount from each hydrogel in different buffer solutions of pH 4.6, 5.4, 6.4, 7.4 and 8.0 at 25 °C for various time intervals until they reached the equilibrium state. After reaching equilibrium, the swelled samples were removed from the media, blotted softly with filter paper to get rid of the surface liquid excess, and reweighed. Equilibrium swelling degree (ESD) was calculated according to the following Equation (2):

(2) $$ESD\;\left(\%\right)=\frac{W_{s}-W_{d}}{W_{d}}\times 100$$

where *W_d_* and *W_s_* are the weights of the hydrogels in dry and swollen state, respectively; each sample’s reported values are the average of at least three measurements.

### 2.7. Biodegradability

The enzymatic degradability of gelatin-based hydrogels (10 mm × 10 mm × 10 mm) by *Clostridium histolyticum* collagenase was evaluated in buffer solution, pH 7.4 (c = 0.1 mg/mL). Each sample was weighed (80 mg) and incubated with 1 mL of the prepared enzyme solution in Eppendorf tubes at 37 °C. Collagenase concentration was comparable to that employed in another, similar experiment to examine the enzymatic degradability of gelatin-based scaffolds for wound management under physiological settings [29]. The mixture was centrifuged at 12,000 rpm for 2 min at predetermined intervals, with the supernatant then being separated for freeze-drying. The hydrogel weight variation was recorded. The percentage of remaining weight was calculated as follows [Equation (3)]:

(3) $$\%remaining\;weight=\frac{W_{t}}{W_{i}}\times 100$$

where *W_t_* is the hydrogel’s weight after being incubated for certain time intervals and *W_i_* is the hydrogel’s weight at the beginning of the experiment. All the experiments were done in triplicate.

### 2.8. In Vitro Loading and Release of Amox from Hydrogels

For the drug loading/release experiments, Amox was utilized.

In all cases, Amox was loaded firstly in nanogels which were dispersed in the hydrogel matrix during their synthesis. MAC5/PAS nanogels’ Amox loading (DL) and entrapment effectiveness (EE) were measured in our prior article [25].

To prevent additional degradation of the MAC5/PAS nanogels or gelatin, which could alter the measurements of the released drug, after weighing each sample (20 mg), they were put in a dialysis bag filled with buffer solution. The pH effect on drug delivery was investigated in solutions simulating the physiological conditions (pH = 5.4 and 7.4, at 37 °C). By taking 1 mL from each release media that was quantitatively assessed by the spectrophotometric method (UV-VIS) at predetermined intervals and replacing it with 1 mL of new buffer solution, the amount of Amox released was examined. Both the drug release rate and the cumulative drug release were determined spectrophotometrically at 231 nm.

### 2.9. In Vitro Cell Biocompatibility Assessment

Alpha-MEM medium (Lonza, Basel, Switzerland) with 10% fetal bovine serum (FBS, Gibco, Thermo Fisher Scientific, Waltham, MA, USA) and 1% penicillin-streptomycin-amphotericin B mixture (10 K/10 K/25 μg, Lonza, Basel, Switzerland) was used to grow the normal dermal fibroblasts (NHDF, PromoCell, Heidelberg, Germany).

The biocompatibility of HGel1, HGel2, HGel4 (unloaded) and AHGel1, AHGel2, and AHGel4 (Amox-loaded) hydrogels was determined by the MTS technique using the CellTiter 96^®^ AQueous One Solution Cell Proliferation Assay kit (Promega, Madison, WI USA) according to the manufacturer’s instructions and ISO 10993-5:2009(E). The cells seed was done in 96-well culture plates at 0.5 × 10^5^ cells/mL. After 24 h, the medium in each well was replaced with 100 μL of hydrogel extract (12.5/6.25/3.125/1.5625 mg/mL) or with fresh medium (Control). Hydrogel extracts were made in complete culture medium at a concentration of 12.5 mg/mL, for 24 h, at 37 °C. Serial dilutions (100%, 50%, 25%, 12.5% extract + complete culture medium) were then made for each analyzed hydrogel. Cells were incubated with extracts for 24 h, then 20 µL of MTS reagent was added per well 3 h before reading the absorbance at 490 nm using a FLUOstar^®^ Omega microplate reader (BMG LABTECH, Ortenberg, Germany). Experiments were performed in triplicate, and the viability of treated cells was expressed as a percentage of the viability of Control cells.

Additionally, the cytotoxicity of HGel4 and AHGel4 hydrogels on normal fibroblasts was determined at concentrations starting from 20 mg/mL (20/10/5/2.5/1.25/0.625 mg/mL in extract 24 h, 37 °C), after 72 h of incubation.

### 2.10. In Vivo Biocompatibility

The assessment of in vivo biocompatibility of gelatin hydrogel was performed after their administration as subcutaneous implants (30 mg). Only the hydrogels HGel2 and HGel4 loaded with Amox were selected (AHGel2 and AHGel4).

For the study, through the UMF biobase Iasi, white Wistar rats, male adults, weighing 200–250 g and having a health certificate from the National Institute for Research and Development Cantacuzino, Bucharest, Baneasa resort, were used.

The animals were housed in plastic cages with free access to water and standard food in rooms with controlled temperature (22–24 °C) and had a consistent 12-h light–dark cycle.

The animals were separated into 4 groups of 6 animals each after a 7-day quarantine period:

Group 1 (M): implant without hydrogels, named negative control (Cn);
Group 2 (SF): implant with cotton pellets soaked in saline solution, named positive control (Cn);
Group 3 (AHGel2): implant with AHGel2 pellet;
Group 4 (AHGel4): implant with AHGel4 pellet.

The effect of hydrogels’ implantation on hematological, biochemical, immunological, and oxidative stress parameters in rats was examined at 24 h and after 7 days in order to evaluate the in vivo biocompatibility of hydrogels.

Leukocyte formula (polymorphonuclear neutrophils or PMN, lymphocytes or Ly, eosinophils or E, monocytes or M, and basophils or B) and liver enzymes (alanine aminotransferase, aspartate aminotransferase, and lactate dehydrogenase or LDH) were evaluated. Additionally, a variety of tests used to evaluate the immune response, including the Nitro Blue Tetrazolium test (NBT), were conducted to look at the activity of serum complement and the phagocytic capacity of peripheral neutrophils [30].

By calculating the activity of malondialdehyde (MDA), the oxidative stress was evaluated. The glutathione peroxidase (GPX) activity was determined by spectrophotometry (DTNB method), high-performance liquid chromatography (HPLC), and the fluorescence detection method. The Randox Ransel Control kit and particular Ransel kits from RANDOX Laboratories Ltd. (Warsaw, Poland) were used to measure the aforementioned parameters. Special analyzers were employed, and the working protocol followed the instructions from each kit.

The experimental protocol was approved by the “Grigore T. Popa” University Committee for Research and Ethical Issues (ethical approval no. 47/15.02.2021), and the research was carried out in accordance with the standards established by the international regulations on the handling of laboratory animals [31].

The experiments lasted for as little time as feasible and utilized the smallest number of animals (6 animals each batch), yet they were nevertheless long enough to provide statistically significant results [32].

Harvested liver fragments were sectioned, fixed in paraffin, and exposed to 10% formaldehyde. A Leica TP 1020 rotary tissue processor (Germany) was used for paraffin embedding, and a manual microtome was used for sectioning (Germany). The blades were created using Masson trichrome staining (hematoxylin-eosin-methylene blue).

An optical microscope (Leica DM 750) equipped with a 3 mpx HD camera and having a LAZ software (2016) was used to examine the preparations.

The experimental data were presented as mean ± standard deviation (SD) of the mean. When compared to the data from the control group, *p*-values under 0.05 were regarded as significant from the statistical point of view.

The one-way ANOVA test and the Tukey test were used to statistically analyze the biochemistry data. Due to these, it was possible to understand the significance of the variations between the hydrogel groups administered subcutaneously and the control group implanted with sterile cotton pellets and saline solution in the same group of animals.

## 3. Results and Discussion

Nano-structured hydrogels have been investigated as a promising strategy to enhance mechanical properties and provide additional stimuli responsiveness. Organic or inorganic nanoparticles can be embedded within hydrogel networks by either physical or chemical interactions and endow hydrogels with enhanced properties like tunable degradation or water affinity capacity for a wide range of biomedical applications. Moreover, these kinds of hydrogels have the ability to be molded and heal themselves after being damaged, due to the dynamic nature of intra- and inter-molecular hydrogen bonds.

### 3.1. FT-IR Analysis

**Figure 1:**
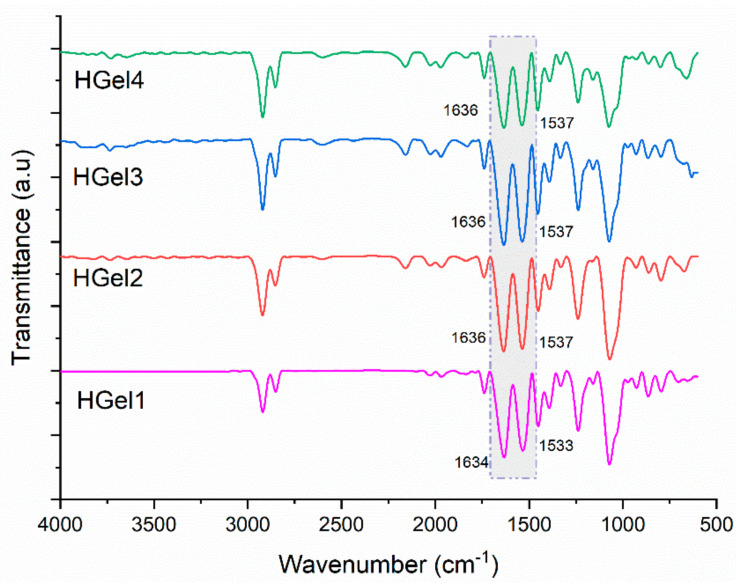
FTIR spectra of gelatin-based hydrogels (loaded with nanogels–HGel2, HGel3 and HGel4 and unloaded–HGel1).

To evaluate the interactions between MAC5 and PAS-based nanogels and gelatin-based enzymatically-crosslinked hydrogels, ATR FT-IR analysis was performed. The IR spectra of HGel1, HGel 2, HGel3 and HGel4 hydrogels are shown in Figure 1. All FT-IR spectra had a similar appearance, showing bands characteristic of gelatin. HGel1 hydrogel containing only gelatin was characterized by peaks assigned to NH bonds at 1533 cm^−1^ and C=O bonds at 1634 cm^−1^. However, in the case of HGel 2, HGel3 and HGel4, the amide I and amide II bands shifted in the presence of MAC5/PAS nanogels up to 1636 cm^−1^ and 1537 cm^−1^, which is an indication of the interaction among the polymers (self-assembled nanogels and gelatin) through hydrogen bonds. This observation is consistent with the results reported in the literature for gelatin and chitosan-based nanocomposites [33].

### 3.2. Morphological Analysis of Hydrogels

**Figure 2:**
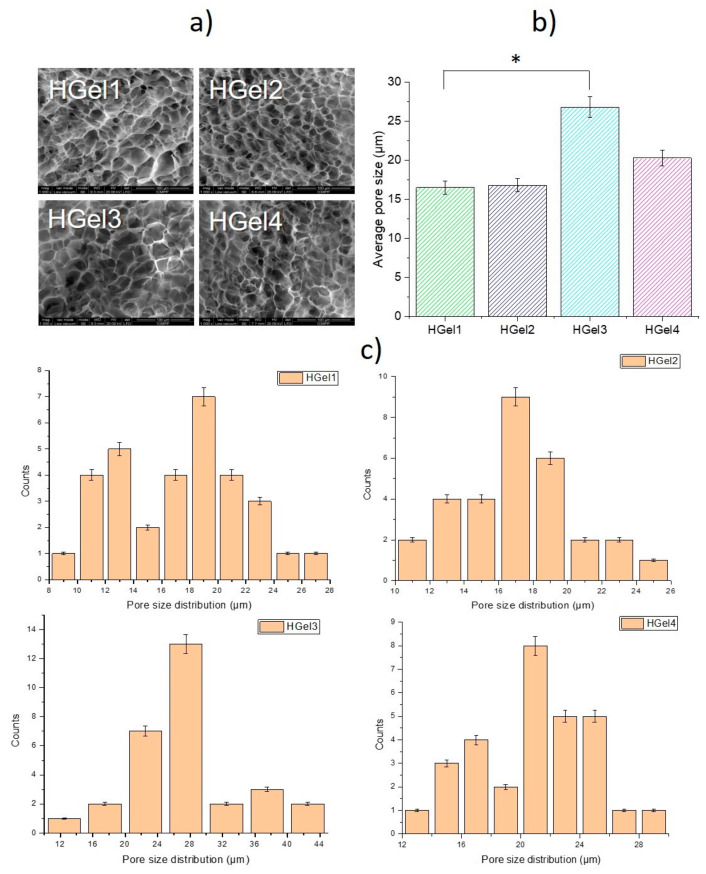
(**a**) Representative SEM images of HGel1, HGel2, HGel3 and HGel4. Scale bar 100µm. (**b**) Average pore size of the hydrogels calculated from SEM images. * *p* < 0.05 by one-way ANOVA with Tukey’s HSD post-hoc test. All statistical analyses were performed using Origin 8.5. (**c**) Histograms of the pore size distribution derived from the cross-sections on the SEM images.

The cross-section architecture of the freeze-dried scaffolds was studied by using the SEM analysis, the results being shown in Figure 2. In the case of HGel1, the recorded SEM images indicated an irregular distribution of the pores in the network, with the internal pore sizes varying from 18 μm to 27 μm. Regarding the morphology of gelatin-based hydrogels loaded with nanogels (diameter pore size between 16–23 μm), the nanocarriers cannot be clearly identified in the pore walls of the hydrogels network, which can be sustained also by the inter-molecular bonds between the interconnected systems. Moreover, in comparison with the simple gelatin hydrogels, it can be observed from the SEM images that HGel2, Hgel3 and Hgel4 composite scaffolds have a homogenous porous network with even distributed pores probably associated with the synergic effect of MAC5/PAS nanogels on gelatin matrix. As expected, after incorporation of MAC5/PAS nanogels, the rigidity of the hydrogel network increased, leading to a slight shrinkage of the pore structures during the freeze-drying process.

Furthermore, HGel3 (27–43 μm) showed bigger pore dimension than HGel1 and Hgel2 (all images at same magnification), and the pore diameter decreased with the increasing of TGase content (HGel4–19–27 μm). In comparison to the simple gelatin hydrogel (HGel1), the average pore size of TGase-crosslinked hydrogel (HGel4) containing nanogels increased, but not statistically significantly. Nevertheless, HGel3 average pore size was associated with an increase that was statistically significant (* *p* < 0.05) compared to HGel1 (Figure 2b). The variation of the pore’s dimensions of gelatin-based hydrogels after nanogels incorporation and TGase crosslinking occurred as a consequence of the interference of the gel-like carriers on the process of gelatin crosslinking. The obtained results are in line with previous results reported in the literature for gelatin-based multiphasic hydrogels incorporating hydroxyapatite particles or cellulose nanocrystals [34].

In medical applications, a highly porous scaffold with interconnected pore network will facilitate nutrients and oxygen exchange and waste removal, and can also highly contribute to cell growth and proliferation.

### 3.3. Swelling Behavior of Gelatin-Based Hydrogels and Self-Healing Capacity Assessment

**Figure 3:**
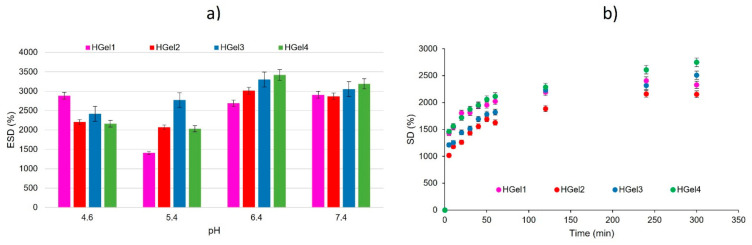
(**a**) Variation of the degree of swelling over time of hydrogels, at pH 7.4. (**b**) Variation of ESD of hydrogel in relation to the change of environmental pH. Means ± SD, n = 3.

The water absorption capacity of the prepared hydrogels in phosphate buffer solution (PBS) solution (pH = 7.4) at 37 °C is presented in Figure 3a. Additionally, the influence of pH on the swelling behavior of hydrogels was evaluated in PBS with the following pH levels: 4.6, 5.4, 6.4, 7.4 and 8.0 (Figure 3b).

It is important to note that hydrogel containing only gelatin (HGel1) swelled considerably and was completely dissolved after 1 day of immersion in buffer solution, over the entire pH range studied. Under the same conditions, hydrogels HGel2, HGel3 and HGel4 kept their stability up to 2–3 days. Moreover, it is observed from Figure 3a that by increasing the concentration of TGase from 2U to 5U in the composite hydrogels, the water retention capacity increased. It may be attributed to the effect of nanogels inclusion in the TGase-crosslinked gelatin hydrogel. The addition of amphoteric nanogels favors the adsorption of water molecules while enhancing the SD of gelatin-based scaffolds [35] and thus, hindering the effect of crosslinking induced by TGase. This observation is in agreement with the one made by other researchers as, for example, the addition of d-sorbitol as a plasticizer increased the water retention capacity of gelatin hydrogels by means of its hydrophilicity [36,37].

As shown in Figure 3a, at pH 4.6, the SD of the hydrogels containing only gelatin was higher compared to the other investigated hydrogels. In the case of composite hydrogels, the nanogels based on modified chitosan and PAS inserted in the hydrogel’s matrix contribute to the decrease of the swelling capacity. This is due to the protonated carboxylic groups of MAC5 distributed on the surface of nanogels [25]. When the pH of the swelling media increases, the water absorption capacity of composite hydrogels increases, causing the network chain relaxation. With the increase of the pH, the incorporated nanogels have a tendency to swell due to the deprotonation of COOH groups originating from functionalized chitosan and PAS. This characteristic is also linked to increased pore sizes of the composite hydrogels (HGel2, HGel3 and HGel4). These pores may result in amplified water transfer to the hydrogel network and increased swelling. This swelling behavior is similar to the one of cress seed gum hydrogel embedding soy protein/sodium alginate nanogel [38].

Figure 3b compares the water absorption capacity of the crosslinked hydrogels after immersion in PBS solution (pH = 7.4) at 37 °C. It can be noted that the swelling capacity of the HGel3 and HGel4 increased slightly due to their less crosslinked networks that result from the hindering effect of MAC5/PAS nanogels on the catalytic activity of TGase.

Based on the absorbance of non-crosslinked and crosslinked hydrogels, when TGase concentration was 2 U/g, the crosslinking degree reached 3.28%, whereas at an enzyme concentration of 4 U/g, the crosslinking degree was 4%. As was observed from the swelling studies data, the inclusions of MAC5/PAS nanogels hinders the isopeptide bond formation between glutamine and lysine in the presence of TGase [39], leading to a lower degree of crosslinking, but enough to control the biodegradability and swelling capacity. 

**Figure 4:**
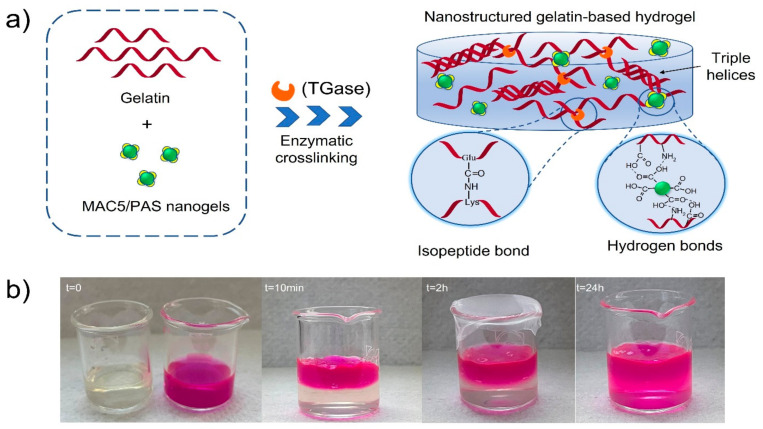
(**a**) Schematic illustration of the fabrication of TGase-crosslinked gelatin hydrogel loaded with MAC5/PAS nanogels. (**b**) Macroscopic self-healing behavior of HGel4 loaded with MAC5/PAS nanogels.

The self-healing capacity of HGel4 was assessed by visual observation and in this regard, images were captured (Figure 4). Therefore, as can be observed from Figure 4, through covalent bonds (enzymatic crosslinking) and physical interactions (hydrogen bonds, electrostatic forces manifested by MAC5/PAS nanogels), a self-healing performance was imprinted to the gelatin-based hydrogels. Despite the fact that each individual bond is weak, together they contribute to the formation of scaffolds with better structural integrity and self-healing capacity that sustain not only the wound healing process through inductive reformation of covalent or noncovalent bonds, but continuously support the reconstruction of the damaged area generated by the multiple cellular processes (homeostasis, inflammation, proliferation, and maturation).

### 3.4. Biodegradability Assessment

Generally, gelatin-based hydrogels used in wound care applications are degraded in the presence of type II collagenase, a member of the matrix metalloprotease family, which degrades the extracellular matrix and stimulates cell proliferation/migration and growth [40]. In this section, the degradability of gelatin hydrogels reinforced with MAC5/PAS nanogels and enzymatically-crosslinked (HGel3 and HGel4) was investigated and compared with the behavior of non-enzymatically crosslinked hydrogels (HGel2). The study was carried out in phosphate buffer solution (pH 7.4) in the presence of collagenase (c = 0.1 mg/mL).

**Figure 5:**
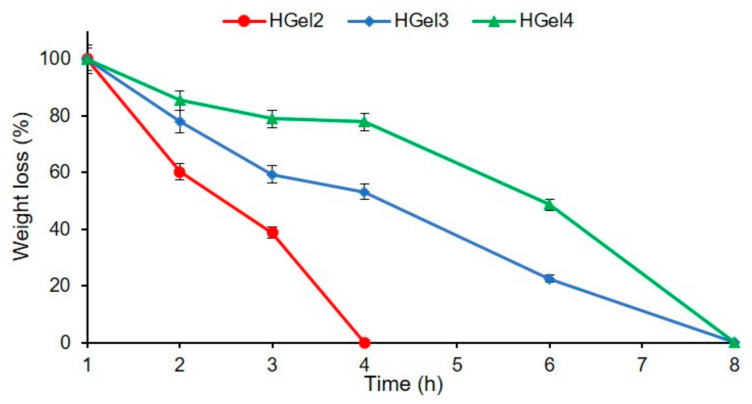
Enzymatic degradation of gelatin-based hydrogels in the presence of 0.1 mg/mL of collagenase, at 37 °C. Means ± SD, n = 3.

During degradation by collagenase, HGel2 hydrogel mass decreased exponentially over time (Figure 5). The hydrogel was completely degraded within 4 h compared to enzymatically crosslinked hydrogels. For TGase-crosslinked hydrogels, the degradation rate was dependent on the concentration of TGase used to crosslink the gelatin matrices. For example, sample HGel3 degraded faster than HGel4, with each preset weighing time having a different degree of degradation. In the first hours, HGel3 and HGel4 hydrogels had a slow degradation rate, which accelerated after a period of time (4 h). These results can be explained by the faster cleavage of gelatin molecules, due to the absorption of a large amount of buffer solution with enzyme. It is most likely that increasing the concentration of TGase or the ratio of nanogels will result in obtaining polymeric scaffolds with controlled biodegradability. However, the tailored biodegradability of these hydrogels can still induce cell proliferation and tissue regeneration, thus reducing the frequency of dressing replacement. These results are consistent with the literature, where physical gelatin-based hydrogels have been reported to degrade faster compared to crosslinked gels (thermo-gelation followed by enzymatic crosslinking) [41].

### 3.5. In Vitro Drug Release

**Figure 6:**
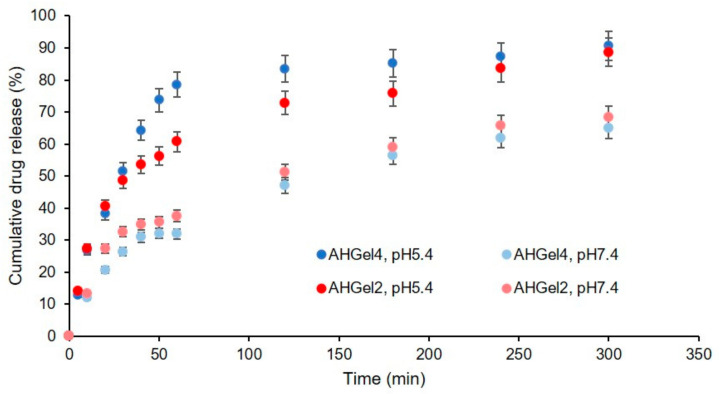
Cumulative drug release of Amox from gelatine-based hydrogel. Means ± SD, n = 3.

In vitro release studies of Amox from hydrogels containing drug-loaded MAC5/PAS nanogels were carried out at two different pH values (5.4 and 7.4) at 37 °C, in a shaking bath. The Amox-loaded hydrogels were coded AHGel2 and AHGel4. The release profiles of Amox from the prepared hydrogels (AHGel2 and AHGel4) are shown in Figure 6. In general, the cumulative release of the drug from the hydrogels is dependent on the swelling of the polymer matrix and solubility of the drug. In the present study, HGel2 and HGel4 hydrogels without Amox presented a higher swelling degree in pH 7.4 buffer solution as compared to the pH 5.4 buffer. Therefore, the drug release should be greater at pH 7.4 as compared to the one in pH 5.4 buffer solution. However, from Figure 6, it can be seen that a higher amount of Amox is released from both hydrogels at pH 5.4 than at pH 7.4. The solubility of Amox can be used to explain the observed trends. It might be due to the fact that Amox dissolves more readily in pH 5.4 buffer than pH 7.4 buffer [42].

Obviously, the incorporation of Amox-loaded nanogels into the gelatin hydrogel had a prolonging effect on the release of the drug, as the three-dimensional network structure of the hydrogel provides an additional diffusion barrier. From Figure 6 it can also be said that the amount of drug released from the investigated hydrogels is not significantly influenced by the enzymatic crosslinking process.

The difference between Amox released from AGel2 and AHGel4 at each pH was low. The percentage cumulative release of drug was 68% for AHGel2 and 65% for AHGel4 at pH 7.4 and 89% for AHGel2 and 91% for AHGel4 at pH 5.4.

### 3.6. In Vitro Cell Biocompatibility Assay

**Figure 7:**
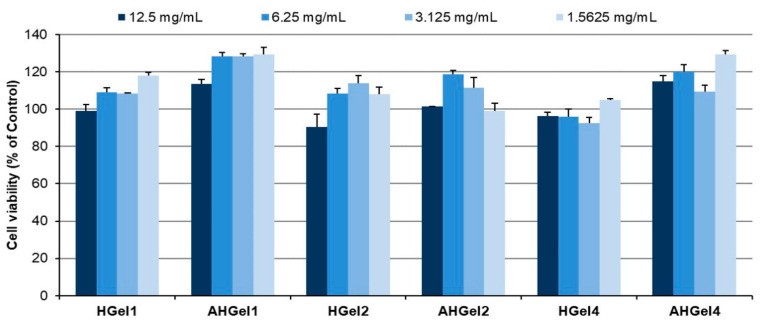
Viability of normal fibroblasts exposed to hydrogel extracts (12.5/6.25/3.125/1.5625 mg/mL) for 24 h. Experiments were performed in triplicate, and the viability of treated cells was expressed as a percentage of the viability of Control cells. Data plotted were expressed as means ± standard error of the mean.

The biocompatibility of HGel1, HGel2, HGel4 and AHGel1, AHGel2, AHGel4 hydrogels was determined by the MTS technique after 24 h incubation with hydrogel extracts (12.5/6.25/3.125/1.5625 mg/mL) or with complete fresh medium (Control). The experimental data showed that extracts from the unloaded hydrogels (HGel) do not influence the viability of normal fibroblasts in culture compared to Control cells (Figure 7). Moreover, compared to Control cells, extracts of drug-loaded hydrogels (AHGel) stimulate the proliferation of normal fibroblasts by up to 29% over 24 h. AHGel1 and AHGel4 stimulate the proliferation of normal fibroblasts over the corresponding unloaded hydrogels (HGel1 and HGel4).

In the case of HGel1 and HGel4 hydrogels, respectively those loaded with Amox, namely AHGel1 and AHGel4, the samples showed a concentration-dependent behavior, the viability increasing with the decrease in the extract concentration. On the other hand, HGel2 and AHGel2 hydrogels had a biphasic behavior, with a slight decrease in cell viability being observed, although the recorded values fall within the biocompatibility range imposed for materials used in biomedical applications. In this case, the optimal concentration of 3.125 mg/mL is the most effective for normal fibroblast proliferation. Most likely, considering that the network formed by the gelatin fibers is not enzymatically crosslinked as compared to the HGel4 and AHGel4 samples, the embedded empty nanogels or ones loaded with Amox diffuse faster, thus influencing the behavior of the fibroblasts and implicitly the values obtained in the cell viability tests.

**Figure 8:**
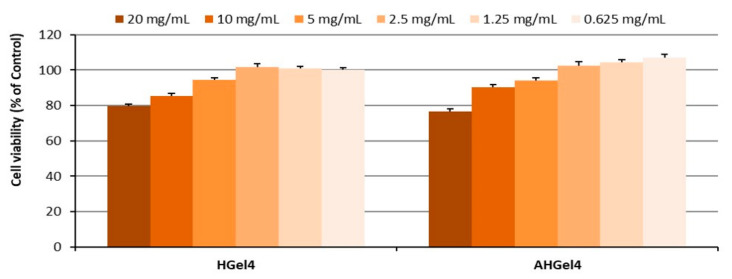
Viability of normal fibroblasts exposed to different concentrations of Gel4 and AHGel4 extract after 72 h. Experiments were performed in triplicate, and the viability of treated cells was expressed as a percentage of the viability of Control cells. Data plotted were expressed as means ± standard error of the mean.

Cytotoxicity of HGel4 and AHGel4 hydrogels on normal fibroblasts was determined at concentrations starting from 20 mg/mL (20/10/5/2.5/1.25/0.625 mg/mL in extract 24 h, 37 °C), after 72 h of incubation (Figure 8). The results showed that HGel4 and AHGel4 are not cytotoxic to normal fibroblasts at concentrations up to 20 mg/mL (<30% mortality). Starting at 2.5 mg/mL, cell viability is maintained at approx. 100% compared to Control cells.

### 3.7. In Vivo Biocompatibility

In light of the use of the gelatin-based hydrogels loaded with MAC5/PAS as scaffolds for biomedical applications, the in vivo biocompatibility of the investigated formulations was evaluated by monitoring the leukocytes formula (polymorphonuclear neutrophils–PMN, lymphocytes–Ly, eosinophils–E, monocytes–M, basophils–B), the activity of liver enzymes (glutamate pyruvate transaminase [GPT], glutamic oxaloacetic transaminase [GOT], and lactic dehydrogenase [LDH]), and immune system parameters (the serum complement level and the phagocytic capacity of peripheral neutrophils [NBT]), after their subcutaneous implantation in rats. Cotton impregnated with saline solution was implanted too, as positive control (Cp). As negative control (Cn), a group without implants was used. The evaluation of some hematological parameters is currently used to assess the level of acute toxicity and the potential to induce inflammation of drug-loading nanosystems.

**Figure 9:**
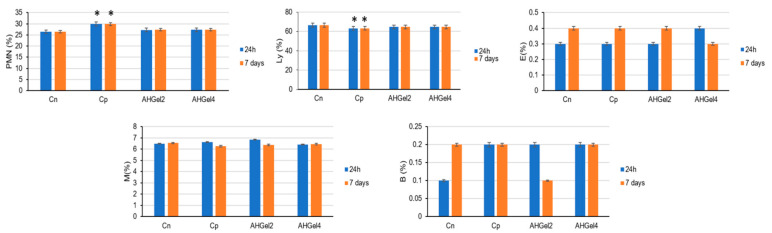
Effects of subcutaneous implantation of Amox-loaded hydrogels on leukocyte formula elements. Values are presented as mean ± S.D. of the percentage of leukocyte formula elements (PMN–polymorphonuclear neutrophils, Ly–lymphocytes, E–eosinophils, M–monocytes, B–basophils) using six laboratory animals per batch. * *p* < 0.05 compared to Cn.

In comparison to the control group without pellets (Cn), the implantation of sterile cotton pellets soaked in saline solution (Cp) was linked with a statistically significant rise in the proportion of PMN in the peripheral blood (* *p* < 0.05) after one day and after seven days. This effect was caused by the inflammatory process that appeared (Figure 9).

At the two time points of the determinations, the subcutaneous administration of pellets containing hydrogels decreased the percentage of PMN in comparison to the cotton pellet impregnated with saline solution group, but not statistically significantly.

In the control animals with granuloma, a statistically significant drop in the percentage of Ly is observed (* *p* < 0.05) versus the negative control group (Cn), after one day and seven days, respectively, in the experiment.

As can be observed from Figure 9, in comparison to the control group with pellets without hydrogels, the percentage of Ly did not significantly change at 24 h or 7 days after implantation when pellets with Amox hydrogels were implanted.

Moreover, there were no appreciable differences between the AHGel2, AHGel4, Cp, and Cn groups in the percentages of E, M, and B at 24 h or 7 days after implantation.

In this study, the percentage values of the components of the leukocyte formula were determined in animals with saline-impregnated pellets, as well as in those administered the amoxicillin-containing hydrogels AHGel2 and AHGel4, and in control animals without pellets. It was found that the implantation of pellets with saline solution produced a local and systemic inflammatory reaction objectified by the significant increase of neutrophils, which are the first cells recruited to the inflammatory site, being the ones predominating in the early stages of this process. However, it was observed that the percentage of neutrophils did not obviously change in the animals in which the pellets with AHGel2 and AHGel4 were inserted, suggesting that these hydrogels loaded with Amox prevented inflammation in the implant area.

**Figure 10:**
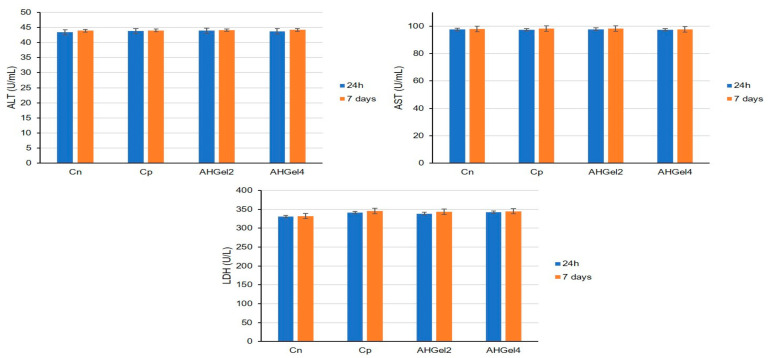
Effects of Amox-loaded hydrogels implantation on ALT, AST and LDH activity. For all data the values are presented as mean ± S.D.

At 24 h and 7 days into the experiment, there were no significant differences between the animals implanted with AHGel2 and AHGel4 hydrogels and those in the Cp and Cn groups in terms of their AST, ALT, or LDH activity (Figure 10).

Following the evaluation of the biochemical profile, no notable differences were highlighted regarding the activity of transaminases and LDH (considered to be good indicators for assessing the functional state of the liver), which makes us appreciate that the administration of the tested substances does not significantly influence the liver function of laboratory animals. These findings correlate with the absence of structural changes in the liver, revealed by the histopathological examination. Histopathological section of the liver of control animals showed normal sinusoids and a central vein surrounded by unaltered hepatocyte architecture. Similarly, animals receiving AHGel2 and AHGel4 showed an unaltered hepatic structure, with no notable abnormalities of the central vein and Kupffer cells.

The ways in which new drug delivery nanoformulations can affect the immune defense capacity of laboratory animals can be investigated through the influence exerted on specific immune parameters, such as serum complement activity and the phagocytosis capacity of peripheral blood neutrophils.

**Figure 11:**
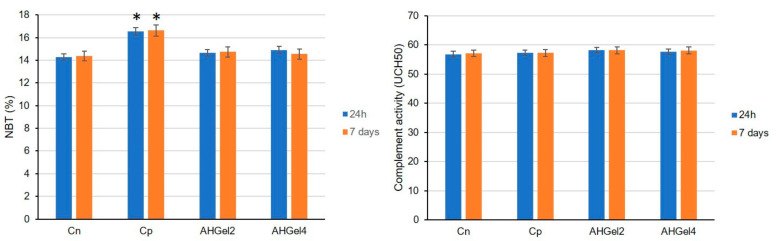
The effects of Amox-loaded scaffolds implantation on PMN phagocytic activity in peripheral blood (NBT test) and on serum complement levels. For all data, the values are presented as mean ± S.D. * *p* < 0.05 versus Cn.

Both after one day and seven days from implantation, the use of saline solution-soaked pellets results in a statistically significant increase (* *p* < 0.05) in the phagocytosis capacity of PMN from the peripheral blood compared to the control group without granuloma (see Figure 11). The body’s reaction to the induced subacute inflammatory process is what causes the effect.

At both time points of the determinations in the study, the application of pellets containing Amox-loaded scaffolds decreased the phagocytosis capacity of peripheral blood PMN compared to the control group with pellets, but without being statistically significant.

No significant variations of serum complement values were found in the AHGel2, AHGel4 or Cp groups by comparison with the Cn group, after 24 h or 7 days after the implantation.

We observed that the phagocytic capacity of peripheral neutrophils and the level of complement in the blood of AHGel2 and AHGel4 rats did not vary considerably during the experiment, being at levels comparable to those of the control group without pellets, which proves that the tested substances do not alter the rats’ immune defense capacity.

**Figure 12:**
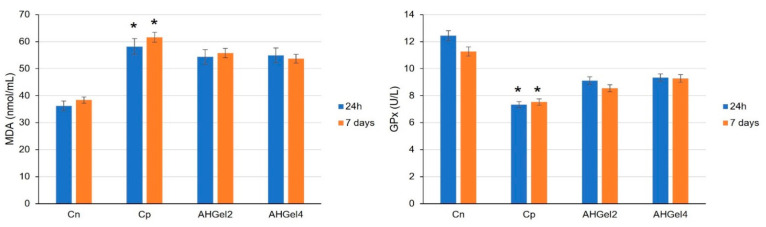
Effects of Amox-loaded hydrogels implantation on serum levels of MDA and GPx. For all data, the values are presented as mean ± S.D. *p < 0.05 reported to Cn.

In comparison to the Cn group, subcutaneous implantation of saline solution-soaked pellets was linked to the statistically significant (* *p* < 0.05) increases in MDA activity and decreases in GPx activity (Figure 12).

Through the utilization of AHGel2 and AHGel4 hydrogel pellets, the MDA values decreased and the ones for blood GPx increased, without being statistically significant by comparison with the positive control group (Cp).

In the Cp group, there was an increase in the serum level of MDA, an enzyme known for its pro-oxidant effects, which reflects an amplified activity of oxygen radicals. At the same time, a significant decrease was found in GPx activity (an enzyme with a key role in the peroxides scavenge) compared to the control group without pellets. All these constitute arguments for the fact that the implantation of subcutaneous pellets resulted in an intensification of oxidative stress. The measurement of MDA and GPx activity showed that the administration of the studied hydrogels significantly decreased cellular oxidation processes in rats.

**Figure 13:**
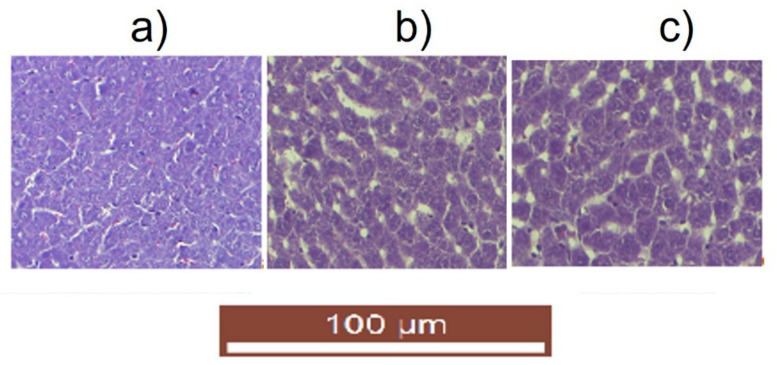
Effects of Amox-loaded hydrogels implantation [(**a**) Cp (**b**) AHGel2, (**c**) AHGel4] on liver histology after 7 days of implantation.

The histopathological evaluation (Figure 13) did not disclose modifications of the liver architecture in the groups that used pellets with Amox-loaded hydrogels (AHGel2 and AHGel4) for subcutaneous administration, and neither in the groups implanted with saline solution-soaked cotton pellets (Cp).

The use of hydrogels containing Amox was not accompanied by significant hematological, biochemical and immune disturbances, and prevented oxidative stress in rats. Additionally, histological evaluation did not reveal relevant changes in liver tissue configuration compared to control animals. These results suggest Amox-loaded hydrogels have minimal toxicity and good biocompatibility, and may be suitable for in vivo use as drug delivery systems, with possible future medical applications, especially on the skin.

## 4. Conclusions

In this study, gelatin-based hydrogels containing Amox-loaded nanogels were prepared through enzymatic crosslinking. The porous structure of freeze-dried hydrogels was characterized by SEM analysis. In comparison with the simple gelatin hydrogels, it was observed that the nanogel-loaded scaffolds have a homogenous porous network with even distributed pores associated with the synergic effect of MAC5/PAS nanogels on gelatin matrix.

The biodegradation rates between simple and nanogels-loaded gelatin scaffolds were significantly different. The degradation rate of gelatin hydrogels was influenced not only by the TGase crosslinking, but also by the inclusion of MAC5/PAS nanogels.

In vitro release of Amox from the gelatin-based hydrogel was examined in pH 5.4 (PBS) and pH 7.4 (PBS) media. Based on the obtained results, decreasing the medium pH from 7.4 to 5.4 accelerates the Amox release from 65–68% at pH = 7.4 up to 89–91% at pH = 5.4 after a period of 300 min.

Furthermore, through in vitro and in vivo biocompatibility assays, all Amox-loaded hydrogels were observed to be biocompatible, even stimulating the proliferation of normal fibroblasts by up to 29% in 24 h. These findings demonstrate that gelatin hydrogels encapsulating MAC5/PAS nanogels have great potential for use in wound care, but also contribute to the inhibition of bacterial spreading through Amox delivery.

## Figures and Tables

**Table 1:** Composition and codification of prepared hydrogels.

Sample Name	Gelatin 4% (mL)	Solution of MAC5/PAS Nanogels (mL)	TGase Concentration (U/g)
HGel1	5	-	-
HGel2	5	1	-
HGel3	5	1	2
HGel4	5	1	5

## Data Availability

Not applicable.

## References

[1] Yang G., Xiao Z., Ren X., Long H., Qian H., Ma K., Guo Y. (2016). Enzymatically crosslinked gelatin hydrogel promotes the proliferation of adipose tissue-derived stromal cells. PeerJ.

[2] Graça M.F.P., Miguel S.P., Cabral C.S.D., Correia I.J. (2020). Hyaluronic acid—Based wound dressings: A review. Carbohydr. Polym..

[3] Diaconu A., Nita L.E., Bercea M., Chiriac A.P., Rusu A.G., Rusu D. (2017). Hyaluronic acid gels with tunable properties by conjugating with a synthetic copolymer. Biochem. Eng. J..

[4] Bostancı N.S., Büyüksungur S., Hasirci N., Tezcaner A. (2022). pH responsive release of curcumin from photocrosslinked pectin/gelatin hydrogel wound dressings. Biomater. Adv..

[5] Xu J., Fang H., Su Y., Kang Y., Xu D., Cheng Y.Y., Nie Y., Wang H., Liu T., Song K. (2022). A 3D bioprinted decellularized extracellular matrix/gelatin/quaternized chitosan scaffold assembling with poly(ionic liquid)s for skin tissue engineering. Int. J. Biol. Macromol..

[6] Furuike T., Chaochai T., Okubo T., Mori T., Tamura H. (2016). Fabrication of nonwoven fabrics consisting of gelatin nanofibers cross-linked by glutaraldehyde or N-acetyl-d-glucosamine by aqueous method. Int. J. Biol. Macromol..

[7] Chou S.F., Luo L.J., Lai J.Y., Ma D.H. (2017). Role of solvent-mediated carbodiimide cross-linking in fabrication of electrospun gelatin nanofibrous membranes as ophthalmic biomaterials. Mater. Sci. Engineering. C Mater. Biol. Appl..

[8] Yue K., Trujillo-de Santiago G., Alvarez M.M., Tamayol A., Annabi N., Khademhosseini A. (2015). Synthesis, properties, and biomedical applications of gelatin methacryloyl (GelMA) hydrogels. Biomaterials.

[9] Vigata M., Meinert C., Pahoff S., Bock N., Hutmacher D.W. (2020). Gelatin Methacryloyl Hydrogels Control the Localized Delivery of Albumin-Bound Paclitaxel. Polymers.

[10] Augustine R., Hasan A., Dalvi Y.B., Rehman S.R.U., Varghese R., Unni R.N., Yalcin H.C., Alfkey R., Thomas S., Al Moustafa A.-E. (2021). Growth factor loaded in situ photocrosslinkable poly(3-hydroxybutyrate-co-3-hydroxyvalerate)/gelatin methacryloyl hybrid patch for diabetic wound healing. Mater. Sci. Eng. C.

[11] Goto R., Nishida E., Kobayashi S., Aino M., Ohno T., Iwamura Y., Kikuchi T., Hayashi J.-I., Yamamoto G., Asakura M. (2021). Gelatin Methacryloyl–Riboflavin (GelMA–RF) Hydrogels for Bone Regeneration. Int. J. Mol. Sci..

[12] Xue X., Hu Y., Wang S., Chen X., Jiang Y., Su J. (2022). Fabrication of physical and chemical crosslinked hydrogels for bone tissue engineering. Bioact. Mater..

[13] Nezhad-Mokhtari P., Ghorbani M., Roshangar L., Soleimani Rad J. (2019). Chemical gelling of hydrogels-based biological macromolecules for tissue engineering: Photo- and enzymatic-crosslinking methods. Int. J. Biol. Macromol..

[14] Sabnis A., Rahimi M., Chapman C., Nguyen K.T. (2009). Cytocompatibility studies of an in situ photopolymerized thermoresponsive hydrogel nanoparticle system using human aortic smooth muscle cells. J. Biomed. Mater. Res. Part A.

[15] Nguyen A.K., Goering P.L., Elespuru R.K., Sarkar Das S., Narayan R.J. (2020). The Photoinitiator Lithium Phenyl (2,4,6-Trimethylbenzoyl) Phosphinate with Exposure to 405 nm Light Is Cytotoxic to Mammalian Cells but Not Mutagenic in Bacterial Reverse Mutation Assays. Polymers.

[16] Choi S., Ahn H., Kim S.-H. (2022). Tyrosinase-mediated hydrogel crosslinking for tissue engineering. J. Appl. Polym. Sci..

[17] Pérez-Rafael S., Ramon E., Tzanov T. (2022). Enzyme-Assisted Hydrogel Formation for Tissue Engineering Applications. Multifunctional Hydrogels for Biomedical Applications.

[18] Jafari H., Ghaffari-bohlouli P., Podstawczyk D., Nie L., Shavandi A. (2022). Tannic acid post-treatment of enzymatically crosslinked chitosan-alginate hydrogels for biomedical applications. Carbohydr. Polym..

[19] Lai E., Bao B., Zhu Y., Lin H. (2022). Transglutaminase-Catalyzed Bottom-Up Synthesis of Polymer Hydrogel. Front. Bioeng. Biotechnol..

[20] Jiang T., Yang T., Bao Q., Sun W., Yang M., Mao C. (2022). Construction of tissue-customized hydrogels from cross-linkable materials for effective tissue regeneration. J. Mater. Chem. B.

[21] Cao C., Feng Y., Kong B., Sun F., Yang L., Liu Q. (2021). Transglutaminase crosslinking promotes physical and oxidative stability of filled hydrogel particles based on biopolymer phase separation. Int. J. Biol. Macromol..

[22] Zhang A., Liu Y., Qin D., Sun M., Wang T., Chen X. (2020). Research status of self-healing hydrogel for wound management: A review. Int. J. Biol. Macromol..

[23] Rusu A.G., Chiriac A.P., Nita L.E., Bercea M., Tudorachi N., Ghilan A., Pamfil D., Rusu D., Cojocaru F.D. (2019). Interpenetrated polymer network with modified chitosan in composition and self-healing properties. Int. J. Biol. Macromol..

[24] Yu R., Yang Y., He J., Li M., Guo B. (2021). Novel supramolecular self-healing silk fibroin-based hydrogel via host–guest interaction as wound dressing to enhance wound healing. Chem. Eng. J..

[25] Rusu A.G., Chiriac A.P., Nita L.E., Rosca I., Pinteala M., Mititelu-Tartau L. (2020). Chitosan Derivatives in Macromolecular Co-assembly Nanogels with Potential for Biomedical Applications. Biomacromolecules.

[26] Chiriac A.P., Nita L.E., Neamtu I. (2010). Poly(ethylene glycol) functionalized by polycondensing procedure with poly(succinimide). Polimery.

[27] Kale R.N., Bajaj A.N. (2010). Ultraviolet Spectrophotometric Method for Determination of Gelatin Crosslinking in the Presence of Amino Groups. J. Young Pharm..

[28] Baseer A., Koenneke A., Zapp J., Khan S.A., Schneider M. (2019). Design and Characterization of Surface-Crosslinked Gelatin Nanoparticles for the Delivery of Hydrophilic Macromolecular Drugs. Macromol. Chem. Phys..

[29] Huang Y.-M., Lin Y.-C., Chen C.-Y., Hsieh Y.-Y., Liaw C.-K., Huang S.-W., Tsuang Y.-H., Chen C.-H., Lin F.-H. (2020). Thermosensitive Chitosan–Gelatin–Glycerol Phosphate Hydrogels as Collagenase Carrier for Tendon–Bone Healing in a Rabbit Model. Polymers.

[30] Lindstrom N.M., Moore D.M., Zimmerman K., Smith S.A. (2015). Hematologic assessment in pet rats, mice, hamsters, and gerbils: Blood sample collection and blood cell identification. Vet. Clin. N. Am. Exot. Anim. Pract..

[31] Directive Directive 2010/63/EU of the European Parliament and of the Council of 22 September 2010 on the Protection of Animals Used for Scientific Purposes. http://eur-lex.europa.eu/legal-content/EN/TXT/?uri=CELEX:32010L0063.

[32] Rollin B.E. (2009). Ethics and euthanasia. Can. Vet. J..

[33] Mathew S.A., Arumainathan S. (2022). Crosslinked Chitosan–Gelatin Biocompatible Nanocomposite as a Neuro Drug Carrier. ACS Omega.

[34] Echave M.C., Domingues R.M.A., Gómez-Florit M., Pedraz J.L., Reis R.L., Orive G., Gomes M.E. (2019). Biphasic Hydrogels Integrating Mineralized and Anisotropic Features for Interfacial Tissue Engineering. ACS Appl. Mater. Interfaces.

[35] Yi J.B., Kim Y.T., Bae H.J., Whiteside W.S., Park H.J. (2006). Influence of Transglutaminase-Induced Cross-Linking on Properties of Fish Gelatin Films. J. Food Sci..

[36] Bakry N., Mohamad Isa M.I.N., Sarbon N. (2017). Effect of sorbitol at different concentrations on the functional properties of gelatin/carboxymethyl cellulose (CMC)/chitosan composite films. Int. Food Res. J..

[37] Bertsch P., Andrée L., Besheli N.H., Leeuwenburgh S.C.G. (2022). Colloidal hydrogels made of gelatin nanoparticles exhibit fast stress relaxation at strains relevant for cell activity. Acta Biomater..

[38] Shahbazizadeh S., Naji-Tabasi S., Shahidi-Noghabi M. (2022). Development of soy protein/sodium alginate nanogel-based cress seed gum hydrogel for oral delivery of curcumin. Chem. Biol. Technol. Agric..

[39] Han Q., Leng J., Dong T., Ma Y., Zhao W. (2022). A Novel Bone Gelatin Prepared by Enzymatic Catalysis with High Crosslinking Activity of MTGase for Gelatinization Properties of Minced Pork. Processes.

[40] Monavari M., Homaeigohar S., Fuentes-Chandía M., Nawaz Q., Monavari M., Venkatraman A., Boccaccini A.R. (2021). 3D printing of alginate dialdehyde-gelatin (ADA-GEL) hydrogels incorporating phytotherapeutic icariin loaded mesoporous SiO_2_-CaO nanoparticles for bone tissue engineering. Mater. Sci. Eng. C.

[41] Lim S., Jeong D., Ki M.-R., Pack S.P., Choi Y.S. (2021). Tyrosinase-mediated rapid and permanent chitosan/gelatin and chitosan/gelatin/nanohydroxyapatite hydrogel. Korean J. Chem. Eng..

[42] Tsuji A., Nakashima E., Hamano S., Yamana T. (1978). Physicochemical properties of amphoteric beta-lactam antibiotics I: Stability, solubility, and dissolution behavior of amino penicillins as a function of pH. J. Pharm. Sci..

